# Supplementary material for: The impact of digital technologies on knowledge networks in two engineering organisations
Source: PLoS One. 2023 Dec 20;18(12):e0295250. doi: 10.1371/journal.pone.0295250 (PMC10732442; doi:10.1371/journal.pone.0295250)
Supplement: S1 Appendix — (PDF) [file pone.0295250.s001.pdf]

Appendix: Semi-structured interview questions

1. Whom would you consult or seek help when you encountered a knowledge gap during work? It'll be very helpful if you could give me all the people you would ask for knowledge.
2. Could you please give me some examples on these consultation situations?
3. Why did you ask him/her/them for knowledge and help?
4. If your colleagues encountered knowledge gaps during work, who would consult or seek help from you?
5. Could you please give me some details on these situations?
6. In the situation of generating new ideas or creating new knowledge, who did, or do you talk with?
7. Could you please give me some examples on these situations? Were these conversations happening mostly informally or formally?
8. How do you normally determine whom to ask for knowledge?
9. Have you ever encountered a situation when a piece of knowledge was very hard to access?
10. Have you encountered difficulties when trying to access the person who processing knowledge you need?
11. What do you think is most important in terms of knowledge management?
12. What are the challenges or difficulties you have encountered related to knowledge management?
13. What are the difficulties or frustrating moments you have experienced related to knowledge management?
14. What are the expected improvements in terms of knowledge management in your daily work?
15. Have you used any digital systems, technologies or tools in your daily work for knowledge management processes? (For example, acquiring, sharing knowledge or triggering new ideas?) What tools? Examples?
16. What digital technologies, tools or support you expect to be involved for knowledge management purpose in your work? Why? Could you please give me some examples?
